# The Prevalence of Small-Pox

**Published:** 1899-03

**Authors:** 


### THE PREVALENCE OF SMALL-POX.

IT IS our desire very briefly to call the attention of the profession to the widespread prevalence of small-pox in the United States, and also to the necessity of vaccination and revaccination as an invaluable means of prevention.

At the present time, according to information gleaned from *Public Health Reports*, the official organ of the Marine Hospital Service, just issued, small-pox prevails to an enormous extent, having invaded twenty-eight states and territories and existing in eighty-nine localities, including the cities of New York and Dunkirk.

However, from our knowledge of it in New York State, it would appear that these statistics only partially represent its prevalence, for a telegram received from Dr. Baxter T. Smelzer, secretary of the State Board of Health, dated February 27, 1899, gives, in addition to the localities named, the following places in this state that have been invaded—namely, Rochester, Penfield, LeRoy, Town Line, Darien and Tonawanda. In all probability there are still other localities afflicted that have not been made known to the state health officials.

Thus the deductions to be drawn from the incompleteness of the statistical record as stated for the Empire State, would be that other states and localities have likewise, in many instances, failed to report its existence to the Federal authorities.

The most efficient means for preventing the spread of small-pox is by vaccination and revaccination. The statistical evidence of the value of this procedure is so voluminous that selection is difficult.

It will be sufficient to quote but a single paragraph from Dr. Bizzozoziro's lecture on the success of vaccination in Germany, delivered at Rome. He said: "Germany stands alone in fulfilling in a great measure the demands of hygiene, having in consequence of the calamitous small-pox epidemic of 1870, enacted the law of 1874 which makes vaccination obligatory in the first year of life and revaccination obligatory at the tenth year. What was the result? With a population of 50,000,000, having in 1871 lost 143,000 lives by small-pox, the mortality diminished so rapidly that today the disease numbers only 116 victims a year."

With pure vaccine lymph from a recognised source, vaccination is an absolutely harmless operation. To insure this, the modern glycerinised lymph only should be used and in this form it is endorsed by the highest authorities known to medical science.

---

THE Christian science fake is now and then in various parts of the country finding itself within the grasp of the law, where, we regret to say, it is too infrequently allowed to escape with inadequate punishment for its crimes. The *Cleveland Journal of Medicine* for January, records one of the latest convictions of a "healer" as follows:

Harriet O. Evans, a "Christian scientist" of Cincinnati, complacently allowed Thomas McDowell to die of typhoid fever without any treatment, except the much-vaunted prayer of these peculiar people. Under the energetic initiative of Dr. Charles A. L. Reed, of Cincinnati, who is a member of the State Examining Board, she was prosecuted for practising medicine without a license. The police-court jury is reported to have employed just twenty minutes in deciding that she was guilty as charged. The case has been appealed, of course, but it is a most excellent beginning. The thanks of the medical profession of the state are due to Dr. Reed for having shown in this and many other cases what a sincere and energetic member of the board can accomplish.

Later advices indicate that a higher court has reversed the decision in this case.

